# Supplementary material for: Complexome profiling of the Chlamydomonas psb28 mutant reveals TEF5 as an early PSII assembly factor
Source: Plant Cell. 2025 Mar 17;37(6):koaf055. doi: 10.1093/plcell/koaf055 (PMC12164590; doi:10.1093/plcell/koaf055)
Supplement: koaf055_Supplementary_Data [file koaf055_supplementary_data.zip › psb28_interactive.zip!/plots/intensity/qualityPlots/peptides/SDH2_Cre06.g264200.t1.2.html]

### Cre06.g264200.t1.2

Trivial name: SDH2  
SeqCoverage [median]: 49.50  
Mass: 32.28 kDa  
  
 

| Sequence | MissedCleavage | Mass [Da] | wtIntensity | muIntensity | Score |
| --- | --- | --- | --- | --- | --- |
| DLVVDMANFYAQYK | 0 | 1675.79 | 29554.12 | 58360.31 | 228.06 |
| EAAKGQEFYQSK | 1 | 1384.66 | 24604.21 | 41472.98 | 147.72 |
| EFQIYR | 0 | 854.43 | 26983.68 | 72345.94 | 149.11 |
| EVDDAYKLYR | 1 | 1270.62 | 769.16 | 2578.80 | 55.57 |
| GQEFYQSK | 0 | 985.45 | 42880.69 | 63141.87 | 194.90 |
| IKDEQDQTLSLR | 1 | 1444.75 | 99809.81 | 177907.58 | 182.55 |
| IKDEQDQTLSLRR | 2 | 1600.85 | 40863.73 | 62109.01 | 126.83 |
| IKQSLAK | 1 | 786.50 | 447.03 | 0.00 | 123.69 |
| MKEVDDAYK | 1 | 1097.51 | 12828.20 | 30159.67 | 112.85 |
| MKEVDDAYKLYR | 2 | 1529.75 | 6929.12 | 14312.47 | 89.96 |
| PAPSRPPLAKPPLYK | 2 | 1630.96 | 61875.33 | 116189.51 | 136.17 |
| PAPSRPPLAKPPLYKEFQIYR | 3 | 2467.37 | 0.00 | 7500.75 | 38.45 |
| SIKPYLQK | 1 | 975.58 | 8802.18 | 28071.27 | 127.12 |
| SIKPYLQKK | 2 | 1103.67 | 1199.13 | 4319.16 | 101.90 |
| VAPLPHMFVVK | 0 | 1236.71 | 89924.06 | 240807.01 | 116.52 |
| VNRDPGHVGK | 1 | 1077.57 | 21755.67 | 34311.76 | 130.01 |
| WIIDSRDDMTSER | 1 | 1622.74 | 4396.89 | 15305.39 | 73.47 |
| WNPDSDEKPK | 1 | 1214.56 | 18121.59 | 37992.54 | 141.89 |
| YLGPAVLLAAYR | 0 | 1305.74 | 2544.11 | 21700.99 | 149.65 |
|  |  |  | 494288.7 | 1028587.0 |  |
